# Supplementary figures and images for: Identifying neural substrates of competitive interactions and sequence transitions during mechanosensory responses in Drosophila
Source: PLoS Genet. 2020 Feb 14;16(2):e1008589. doi: 10.1371/journal.pgen.1008589 (PMC7173939; doi:10.1371/journal.pgen.1008589)

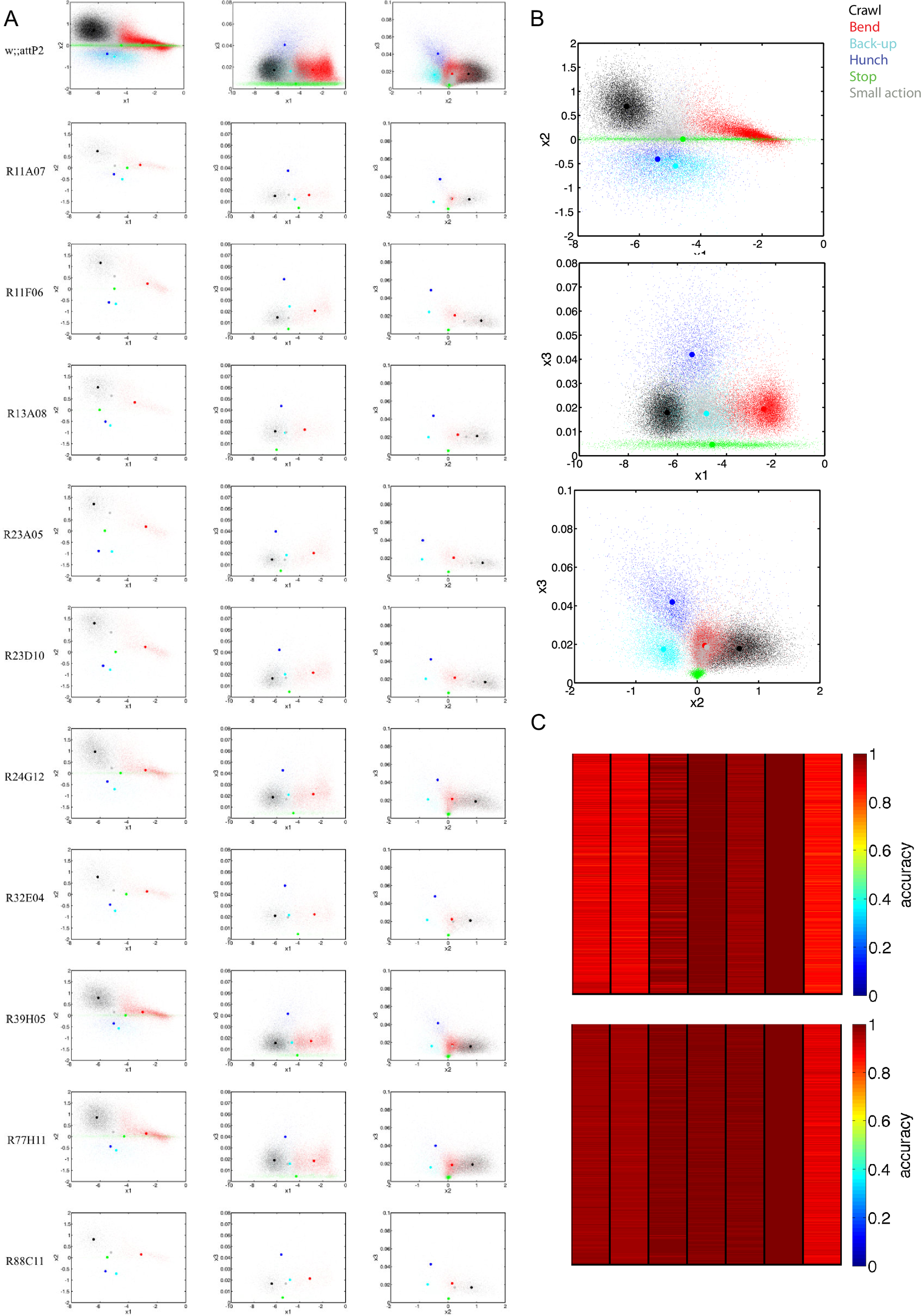

Supplement: S1 Fig — A. Scatter plots of 3 larva features with 3 projections for the control and 10 hit lines (plotted per behaviour). Each point is the average value of a larva feature when performing one of the behaviour during one experiment. Colour code is associated to behaviour with: in black Crawl, in red Bend, in Green Stopped, in blue Hunching, in Cyan Back-up and in grey-small motions. The plot was performed on ca 13000 larvae from the w::attP2-TNT experiments. The features shown here are x1=log((1−〈S〉10)2),x3=〈v〉10〈l〉25α10,5 and x4=〈(dldt)2〉10. Note the natural separation of the features with the behaviour; small motion lies in between Crawl, Bend and Stop. Hunch and Back-up are close together. You can also note the difference in the geometry of the features linked to action. B. Same as in A for the control plotted per larva C. Accuracy of behavior identification with limited features with in knn classifier (top panel) and in random forest classifier (bottom panel) (TIF) [file pgen.1008589.s001.tif]

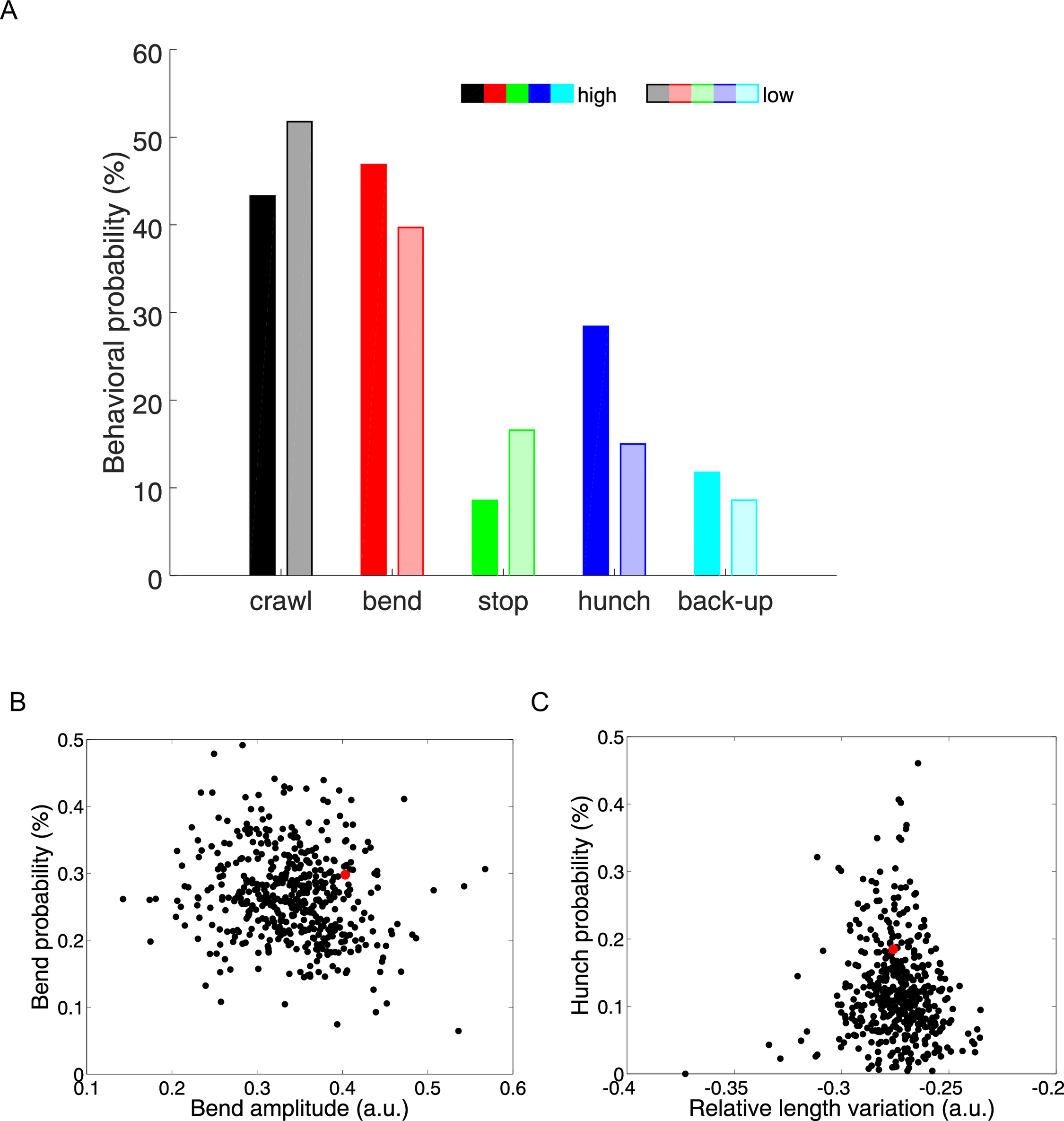

Supplement: S2 Fig — A. Behavioral probabilities upon stimulus onset (during the 1st second of stimulation at high (6 m/s) and low (3 m/s) intensities of air-puff. p-values are all p<0.001 (***). B. Scatterplots of Amplitude of Bending (effective angle) vs Bend probability. C. Scatterplots of Amplitude of Hunching (Relative length variations) and Hunch probability. B-C. Each dot represents the average value for a neuronal line. (TIF) [file pgen.1008589.s002.tif]

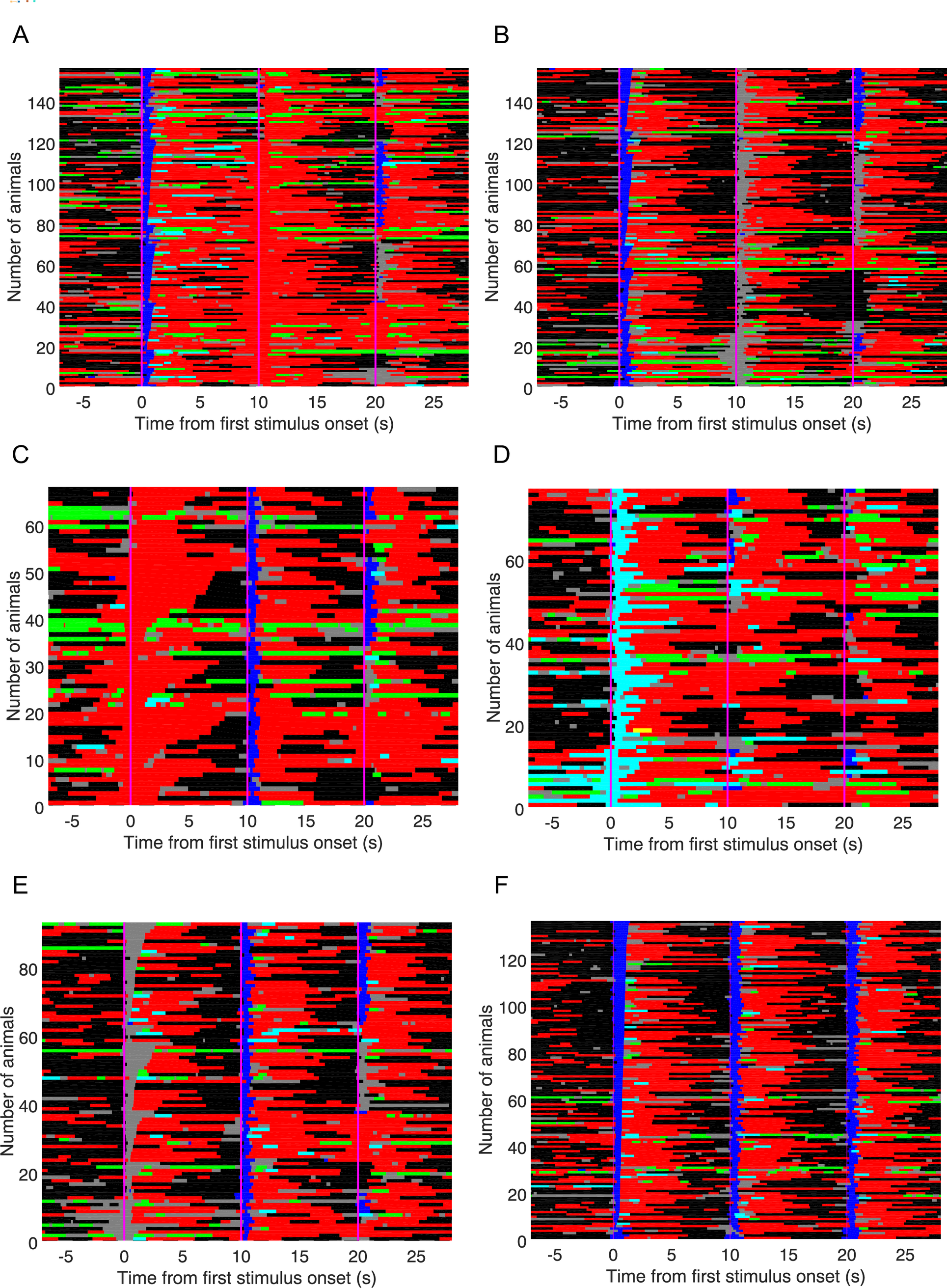

Supplement: S3 Fig — A-F. Ethogram of the behaviors of the attP2>TNT control before, and upon repeated presentation of 2 s air-puff stimulation. Each line is a larva. Different colors represent different actions: Blue-Hunch, Red-Bend, Cyan-Back-up, Black-Crawl and in Green-Stop A-E. Different examples of inter-trial variability (within each ethogram) and inter-individual variability (between different ethograms). A. Example of larvae that hunched on the first stimulus, bended on the second stimulus and performed any action on the third stimulus. B. Example of larvae that hunched on the first stimulus, performed a small action on the second stimulus and performed any action on a third stimulus. C. Example of larvae that bended on the first stimulus, hunched on the second stimulus and performed any action on the third stimulus D. Example of larvae that backed-up on the first stimulus, and performed any action on the second and third stimulus E. Example of larvae that performed a small action on the first stimulus, hunched on the second stimulus and performed any action on a third stimulus F. Example of larvae that only hunched immediately upon repeated presentation of an air-puff stimulus (TIF) [file pgen.1008589.s003.tif]

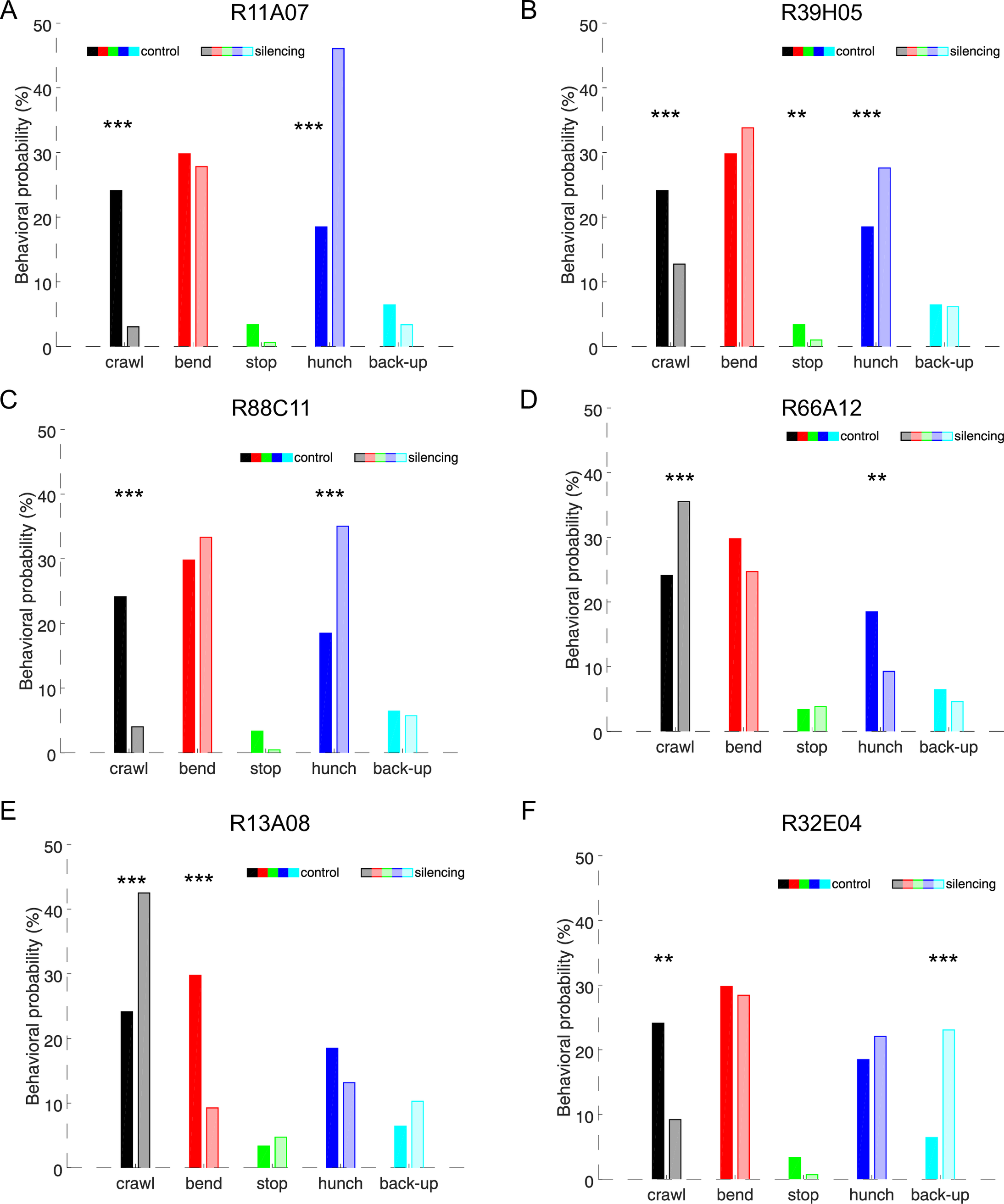

Supplement: S4 Fig — A-F Behavioral probabilities upon stimulus onset (during 1st second) for all the selected lines shown in Fig 4. for the five behaviors: Crawl, Bend, Stop, Hunch and Back-up are shown l *:p<0.05, **:p<0.01, *** p<0.001 A. R11A07, B. R39H05, C. R88C11, D. R66A12, E. R13A08, F. R32E04. (TIF) [file pgen.1008589.s004.tif]

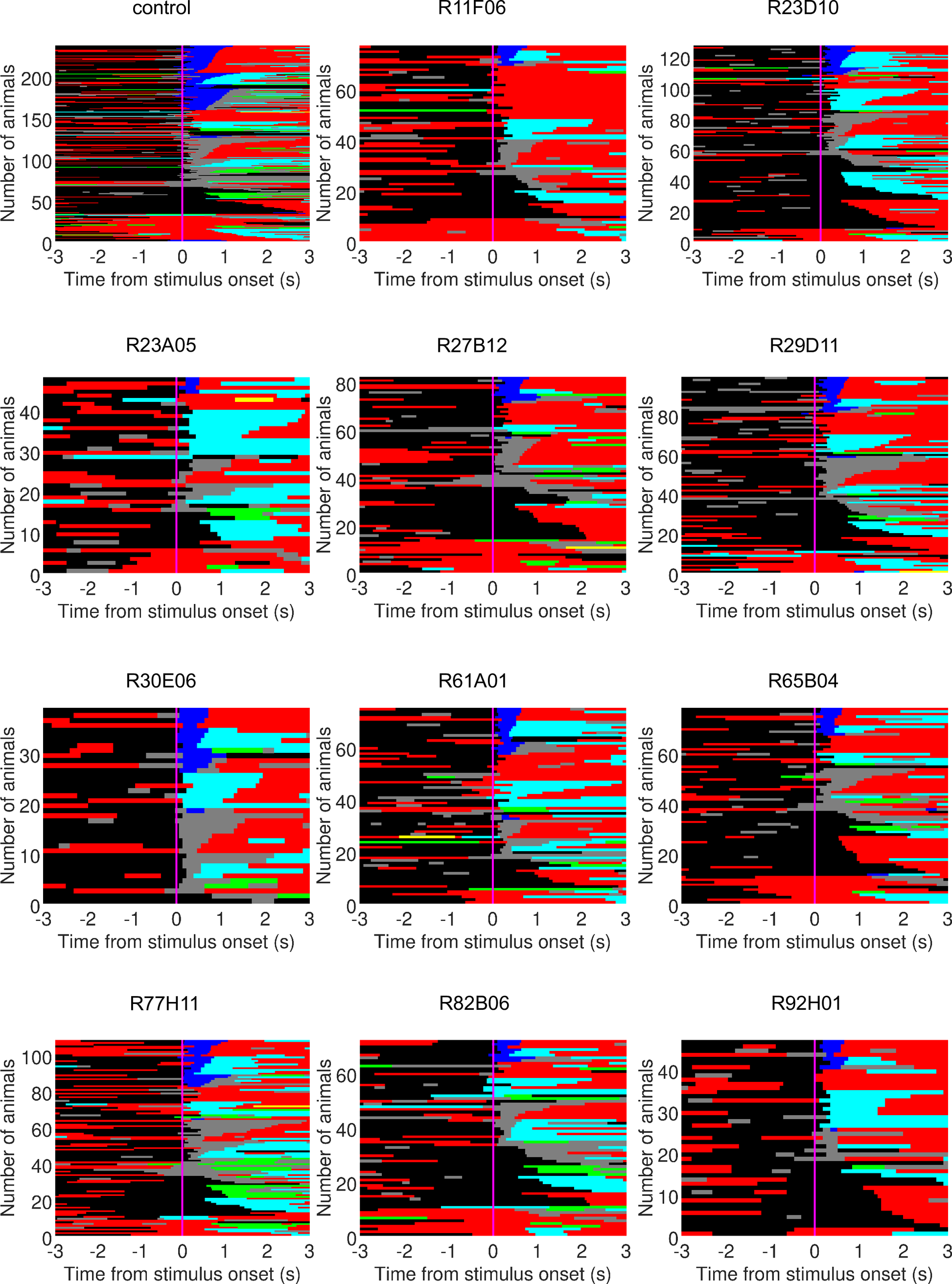

Supplement: S5 Fig — Ethograms for all the selected competitive interaction hit lines with sparse neuronal expression patterns shown in Fig 6. (TIF) [file pgen.1008589.s005.tif]

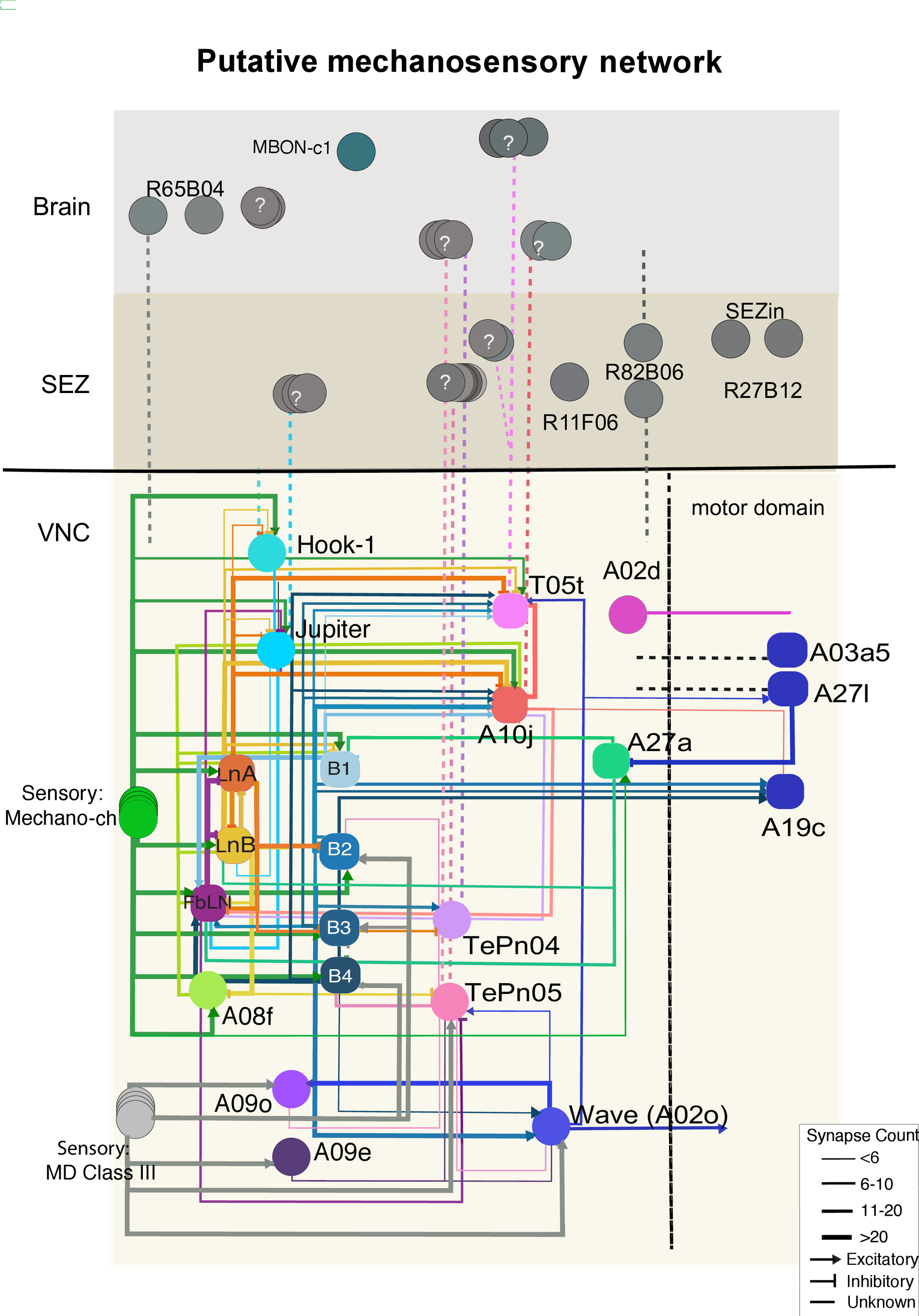

Supplement: S6 Fig — –a summary diagram of all the neurons identified in the hit lines as putative neurons involved in mechanosensory responses in this study and some previously published key neurons involved in mechanosensory responses: Basin-1-4 (B1-B4), LnA, LnB, fbLN, A09e [10,21,38]. (Their detailed connectivity can be found in [10,21,30,38]). Connectivity is based on previously published reconstruction and newly reconstructed connections (S1 Data file, connectivity matrix). The arrows indicate excitatory connections, T-bars indicate inhibitory connections and plain lines when it is unknown whether it is excitatory or inhibitory. The width of the line is proportional to the strength of the connection. Some weak connections are not shown for clarity the number of synapses may be underestimated due to the lack of data from segments. Putative connections are shown as dashed lines. In addition to the reconstructed network in the VNC, putative neurons in the SEZ and brain are shown based on the candidate neuron from the behavioral screen (i.e. lines R92H01, R11F06, R82B06, R65B04, R27B12) (TIF) [file pgen.1008589.s006.tif]

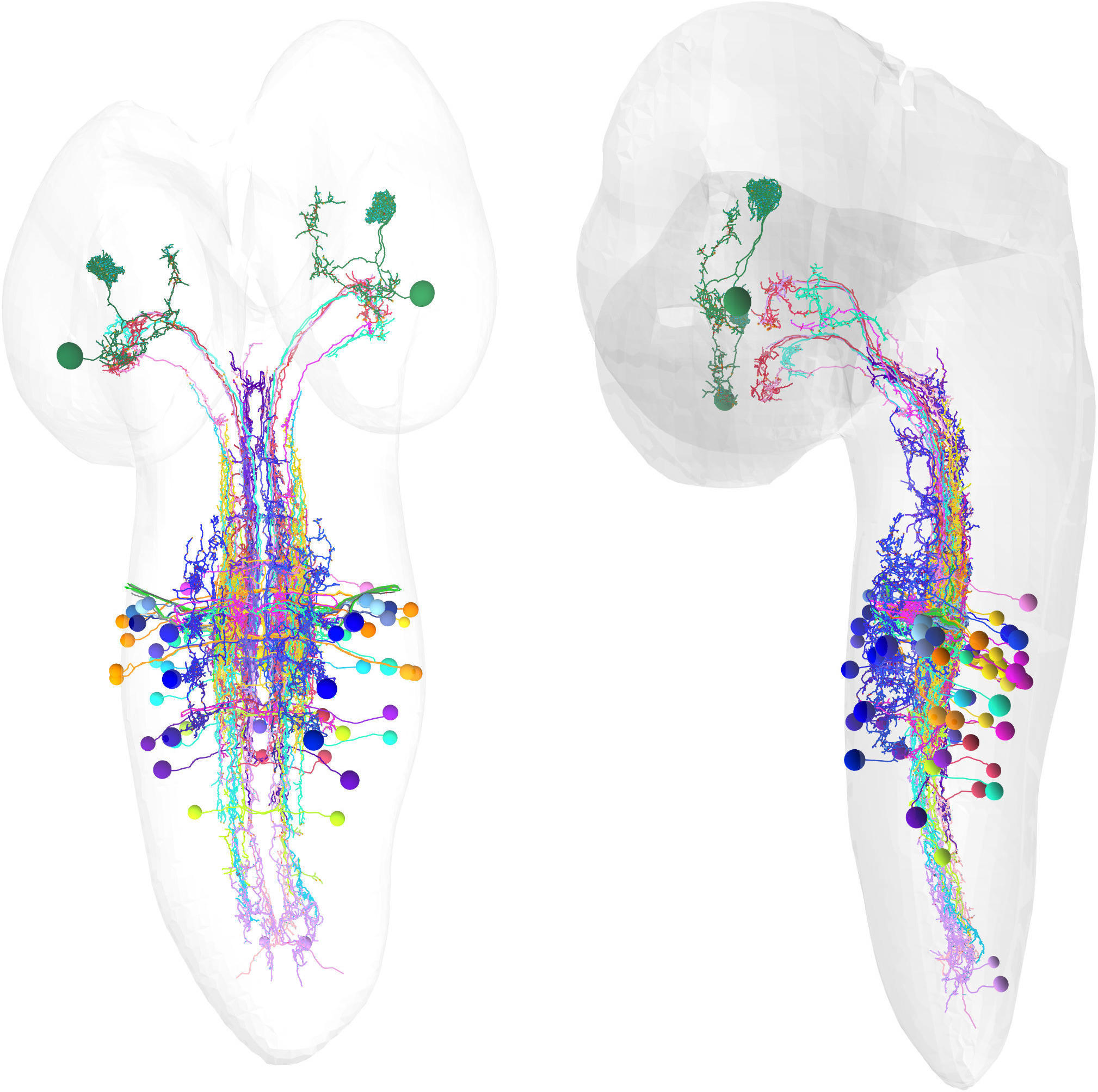

Supplement: S7 Fig — All the elements from the putatitive mechanosensy network shown in S6 Fig. Dorsal view (left panel), Lateral view (right panel). (TIF) [file pgen.1008589.s007.tif]

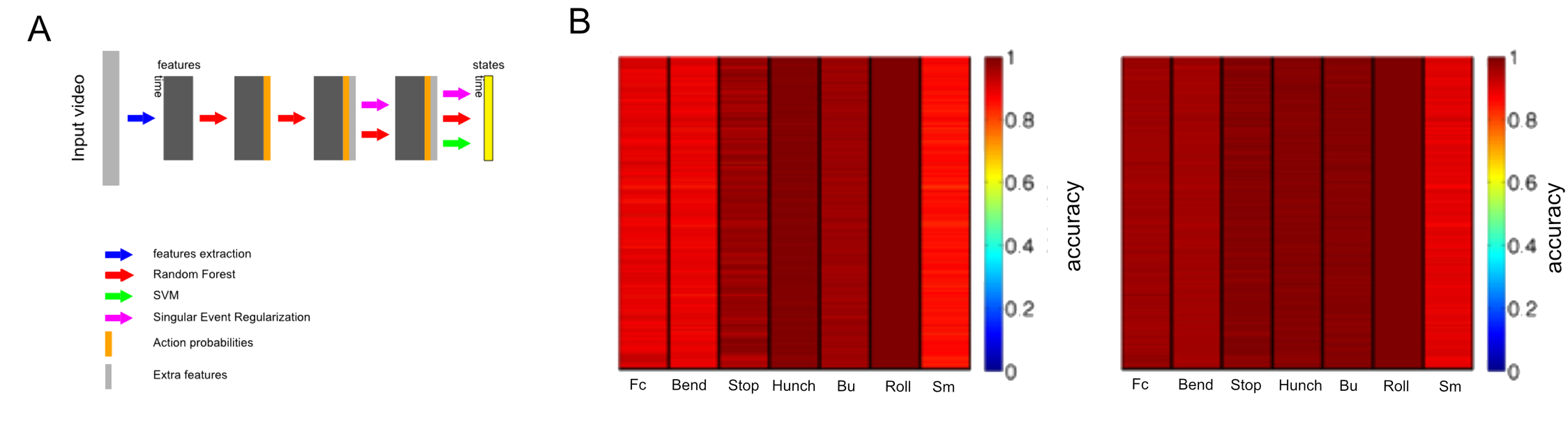

Supplement: S8 Fig — A. Graphical representation of the learning procedure. B. Accuracy of behavior identification with limited features with on the left, knn classifier and on the right, random forest classifier. Every line is a GAL4 line and every column is an action: Fc is Forward Crawl, B-u is Back-up and Sm is Small motion. Note the overall constant accuracy throughout the lines. (TIF) [file pgen.1008589.s008.tif]

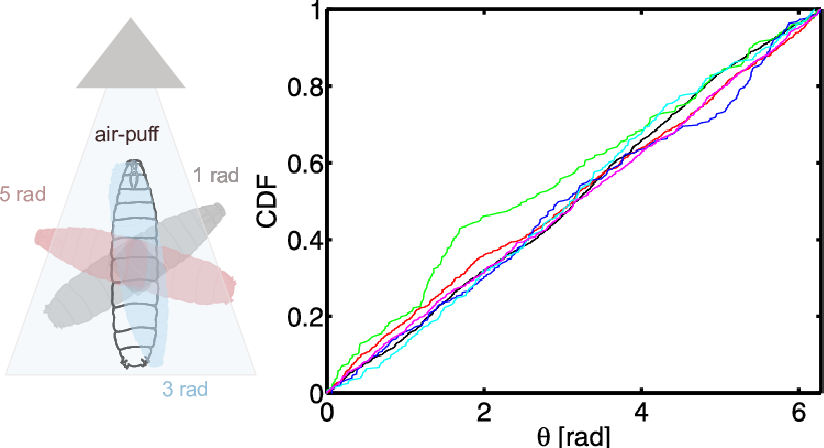

Supplement: S9 Fig — Evolution of the Cumulative Distribution Function (CDF) of the angle θ=θe1→−θw with. θe1→ the absolute angle of the e1→ vector (top neck to head of the larva) and θw = 3π/2 is the angle associated to the wind direction, with the first action after the air puff stimulus. Color code is associated with behavior (similar to all other plots in this paper) with small actions in purple. Note that linear evolution of the CDF is associated to flat (no preferred direction) of the probability density function (pdf). (TIF) [file pgen.1008589.s009.tif]
