# Supplementary material for: Identifying neural substrates of competitive interactions and sequence transitions during mechanosensory responses in Drosophila
Source: PLoS Genet. 2020 Feb 14;16(2):e1008589. doi: 10.1371/journal.pgen.1008589 (PMC7173939; doi:10.1371/journal.pgen.1008589)
Supplement: Attachment — Submitted filename: Masson PLoS Genetics 2019.pdf [file pgen.1008589.s026.pdf]

I recommend for publication in PLoS  Genetics " Identifying neural substrates of competitive interaction and sequence transitions during mechanosensory responses in Drosophila" by Masson et al. 2019

In this manuscript the authors describe the results of a behavioral screen. First, they describe wild type behavior in response to air-puff stimulus. Then they describe the results of a behavioral screen, in which hundreds of GAL4 lines are used to drive tetanus toxin to constitutively silence small subsets of neurons. Notably, the phenotypes described in this study were detected using their new automated action detection pipeline. The authors characterize several classes of behavioral phenotypes: 1) those seen upon silencing sensory (and other) neurons, 2) upon silencing "first order" and pre-motor interneurons, and 3) lines whose silencing results in more complex phenotypes— either competative interaction phenotypes or transition probability phenotypes. Finally, the show a map of the know connectivity among lines they describe.

My opinion is that this is an incredibly rich dataset, and its publication will provide an important resource for the field.

I have a number of minor comments that should be address before publication to improve readability of the manuscript. Below.

1) The figure legends are rife with typos.
2) line 113. Is this a re-analysis of a pre-existing dataset?
3) In Figure 2, panels B-D need further explanation either in the text or the Figure legend. These plots are not standard for the field. What does each black dot represent, red dot? Do plots B-D correspond to categories showing in E? The axis font is very small. This is true for many plots.
4) line 186. Would be useful to state where in the text the hits for competitive interactions will be discussed, where in the text hits for other phenotypes will be discussed, etc.
5) Figure 3. Heatmap key for E-G blue and red both code for decrease? This doesn't seem to match with supplementary tables, or make sense. True also for Figure 5.
6) line 234. totally unclear why the authors bring up ES neurons here.
7) line 260-269. Repetitive.
8) line 275, 342. What is the measure of sparse? Earlier the authors stated all lines were pre-selected for sparse expression (Figure 2A).
9) Figure 4. not all plots are label, e.g., order of plots does not follow text, or any discernible order.
10) line 277. Distracting to mention R32E04 here.
11) line 277. Before discussing Ladders, Basins, and other connections a brief 1-2 sentences of known players in anemotaxis and Hunching would give the reader needed context to understand the connectivity discussed in this section. Otherwise, it reads like a meaningless laundry list.
12) In general sections 2.4 and 2.5 are difficult to read and need to be re-worked with more context. I recommend moving the connectivity into a figure panel and discuss the implications of the connectivity in the main text rather than provide a long list that give the reader little insight.
13) lines 352-361. You cannot write a sentence like this and expect the reader to follow it.
