# Supplementary material for: Identifying neural substrates of competitive interactions and sequence transitions during mechanosensory responses in Drosophila
Source: PLoS Genet. 2020 Feb 14;16(2):e1008589. doi: 10.1371/journal.pgen.1008589 (PMC7173939; doi:10.1371/journal.pgen.1008589)
Supplement: Attachment — Submitted filename: Response to reviewers.pdf [file pgen.1008589.s027.pdf]

Responses to Reviewers' Questions

We would like to thank the reviewers for their positive views of the paper and their questions. You will find in the following in blue our responses, in black the questions and remarks from the referees and between quotation marks portions of the text that have been added/modified.

Reviewer #1: The review is uploaded as an attachment.

1) We have corrected the typos in the figure legends

2) These data have not been published before

3) We have increased the font on the axes of the plots in 2B-D and elsewhere in the manuscript where appropriate (especially in Figure 1, 4 and 5). We have also clarified in the legend for Figure 2 what each for dot and different phenotype categories represent. Indeed, phenotype categories shown in Figure 2 E were determined based on the phenotypes in Figure 2 B-D. We have added a sentence that points that out in the text and this section now reads (starting at line 277):

   "We then examined the different types of phenotypes (Fig. 2 B-D, S1 Table) and found group of hits where: 1) one or more actions decreased, 2) one or more actions increased compared to the control and 3) one action was increased while (at least) another was decreased compared to the control. Based on these we determined three phenotype categories: 1) less actions, 2) more actions and l3) less of one action (or several actions) and more of another (or several other actions)."

4) We have added a sentence that states where these categories will be discussed (l. 293)

5) We thank the reviewer for noticing this. We have corrected this in both Figures 3 and 5

6) We have removed the part of the sentence referring to external sensory lines and the sentence now reads:
   "We obtained a line that labels multidendritic class III (md III) neurons selectively" (l. 352 in the text)

7) We have shortened this paragraph

8) We first selected the lines for screening (567 lines) based on quality of expression as well as sparseness (less then 10 neuron types labeled per segment). In some cases when an intersectional strategy could be envisioned to narrow down the expression pattern, for example by limiting the expression only to the brain lobes or the VNC broader lines were also selected. After hit detections based on phenotypes in

behavioral probabilities, we further selected to analyze in detail the hit lines with very sparse expression patterns: one to four neurons (line 397)

9) We thank the reviewer for noticing this. We labeled all the plots. The plots were arranged so that all the lines with phenotypes in hunching were grouped in one line (bottom) and the lines with phenotypes in bending and backing-up are in the top line.

10) We have removed the part of the sentence referring to R32E04 and it now reads:
"We used multicolor flip-outs to morphologically characterize the neurons in the lines whose silencing resulted in more Hunches (i.e. R11A07, R88C11, R39H05) (Fig. 4 D-F)."

11) We have added a paragraph in the section 2.4. to provide context for the neurons that relate to the hunching and bending as well as anemotaxis (l.403-433)

12) We have provided more context for the previously identified circuitry in sections 2.4 and 2.5 and clarified the description of the connectivity. We have also replaced the Figure 8 with the simplified representation on the connectivity based on sections 2.4. and 2.5. that reveals parallel ascending pathways (to higher regions of the nervous system) as well as to the motor regions. The old Figure 8 is now supplementary Figure S6.

13) We have made this section clearer, by making shorter sentences and it now reads:

In the R61A01 line we identified four different neurons: A10j, A09o, TePn04 and TePno5. The A10j neuron (Ohyama et al., 2015) receives input from LnA (Drunken-1, Griddle-1,2) LnB (Ladder-a) and Basins neurons and synapses strongly on the T05t neurons and the FbLN (feedback interneurons) that are involved in sequence transition and maintenance (Jovanic et al., 2016). The A09o neuron (Gerhard et al., 2017) receives inputs from multidendritic class III and Wave neurons (Ohyama et al., 2015; Takagi et al., 2017) as well as projections neurons along the nerve cord. The TePn04 neuron (Ohyama et al., 2015), receives input from Basin-2 and LnA and LnB neurons and ascends to the higher order regions while the Tepn05 neuron receives input from multidendritic class III (and IV) sensory neurons and related interneurons (i.e A09e, DnB, Wave (Burgos et al., 2018; Jovanic et al., 2019; Takagi et al., 2017), as well as FbLN neurons and synapses onto Basin-2 and 4, the Wave and A09o neuron and projects to higher order regions (Burgos et al., 2018; Gerhard et al., 2017).

Reviewer #2: Review of Masson et el PGENETICS-D-19-01524

Masson et al describe the results of a circuit screen that was carried out using larvae of Drosophila melanogaster. 567 Janelia GAL4 lines were pre-selected for anatomically sparse expression patterns in the larval brain and/or peripheral nervous system. These lines were then crossed to a UAS-TnT to silence the neurons and larvae with the silenced neurons were exposed to an air puff stimulus. Video recordings were made of the animals with the silenced neurons before, during, and after responding to the stimuli,

and the behavioral responses were analyzed using a supervised machine learning approach. Five general categories of behavioral responses are seen in various probabilistic combinations in response to an air puff stimulus (hunch, crawl, bend, stop, backup). By comparing the responses of the silenced animals to controls, the authors identified GAL4 lines that resulted in alterations in behavioral probabilities. Neurons targeted by these lines were then described and investigated further. Neurons identified were in the peripheral nervous system and distributed throughout the brain. The authors conclude that the results are consistent with a "distributed model of decision making." The paper is very rich in data, and the experiments are well-performed, and the study will be of interest to the readers of PLoS Genetics. However, the conclusion that the data support a distributed model of "decision making" is overstated and this needs revision and further discussion

Major Points:

1.) The methods contain a very detailed discussion of the mathematical parameters used in the behavioral classification scheme, but there is almost no discussion of this in the text. The manuscript would be improved by a short discussion on the types of errors that can arise from this analysis. Are there caveats that the readers should be made aware of?

We thank the reviewer for pointing this out. We have added a paragraph in the results section that gives more details about the method (l.165-191).

"We first characterized the response of intact larvae to a mechanical stimulus: air-puff. We used a supervised learning algorithm that relies on a limited set of features associated to the shape, velocity and dynamical evolution of associated variable. The method was trained iteratively on a small number of larvae annotated manually, and is able to handle low resolution images of larva to specifically detect five action categories that we observed in response to air-puff: Hunch, Stop, Bend, Back-up and Forward Crawl. It can also detect a sixth action (Rolling), that doesn't typically occur in response to air-puff and is not a subject of this study. The actions are mutually exclusive and non-overlapping (S1 Fig.). In order to ensure that this representation was stable in different genotypes and individual differences across the screen we applied the clustering to selected tested lines (S1 Fig.). The stability of the representation throughout the screen (as well as in the lines of interest as seen in figure S1) suggest that representation of behavior as discrete action is not dependent on experimental conditions or on subset of larvae sharing common (unknown-unseen) properties. This natural clustering allows then associating to them various relevant amplitudes (velocity, angles of various part of the body, body length, derivatives of body length etc.) characterizing the action through continuous variables. Actions are also characterized by duration as the time spent within on cluster and naturally transitions from previous action to the current one and transitions from the current one onto the next. This description of behavior allows separating nature of actions from all properties associated to them and thus allows probing selection processes during competitive interactions. Furthermore, within the current experimental paradigm, this description allows capturing the sequence of action generation where each discrete action has the same definition for all larvae. We equality minimized the effects of other factors on detection accuracy. For example, the tracking quality may affect the accuracy of detection (i.e. if the larva is tracked for very short periods of time and therefore tracks shorter than 20s were excluded from the analysis (see Material and

methods). Some actions may be ambiguous when they have very small amplitudes and durations and are therefore according to the definitions of actions used in this study fall in the small action category and therefore do not bias any of the categories. More details about the behavioral detection method can be find in the material and methods section of the paper including the unsupervised scoring of the approach allowing us to quickly evaluate the quality of the behavioral dictionary (S1C Fig)."

In Figure S1C we show an unsupervised scoring of the approach allowing us to quickly evaluate the quality of the behavioral dictionary. Stability over the entire screen enforce the possibility of analysis at the screen scale. Similarly, in Figure S1A, we show for lines of interest the embedding with the assigned states. Here again, both the clusters shape and position remain unchanged for all lines suggesting both the existence of this basis and its stability for large amount of larva. Our current approaches only involve unsupervised learning method (that do not rely on manually tagged data for training) to build enhanced behavior dictionaries. We still rely on the current algorithm to assemble datasets where identified behavior will be represented and which will be used for unsupervised approaches.

2.) The authors should justify their use of the term decision making. Although the term "decision making" is thrown around broadly in the field of biology, when it is used in the context of animal behavior and ethology there is a very specific meaning. In the context of behavior, the term "decision making" should be reserved specifically for when the organism is making choices between options that have quantifiable costs and benefits.

It is true that the term "decision" is more loosely used in the field of neuroscience in other contexts. For instance, during development, one can say that an axon makes a decision to either be repelled or attracted in a morphogen gradient. It can also be said that an enhancer "makes a decision" to be activated or repressed depending on the constellation of transcription factors that are present in a cell. A neuron makes a decision to fire or be repressed from firing depending on the balance of excitatory and inhibitory inputs that it receives.

But using the term decision making in the field of neuroethology necessitates that there is utility in the choice. A decision involves weighing costs and weighing the quality of the information that is being used in assigning values to the choices. These ideas are embodied and clear with valid uses of the term. A fly makes a decision when it is either attracted or repelled by an odor depending on whether or not this odor has been previously paired with an appetitive or an aversive stimulus. Or an odor can be attractive or repellent based on innately (evolutionarily) determined costs and benefits. A fly decides to lay an egg or to hold an egg depending on the complexities of the sensory inputs at the oviposition sites. The energetic resources involved with producing the egg are a clear cost to making an incorrect decision.

Thus, it can be argued can whether or not the larval behaviors under investigation involve genuine

decision making. What is the cost to the larvae of producing one behavior or another in response to the air puff? What is the benefit? While it is clear that the neuronal network in the larval brain will produce a variety of behavioral outcomes if given an apparently identical stimulus, it is less clear that this is the result of a decision. The probabilistic nature of the response makes it more akin to the rolling of dice than to making a decision.

It is logical that the network that receives this body-wide sensory input would encompass neurons that are distributed across the brain, the thoracic, and the abdominal ganglia. However, it is not logical to conclude from this observation that the seat of "decision making" for this behavior is distributed across the brain. It is especially not appropriate to extend this finding to the rules of decision making in general. These interpretations of the results are overstated.

It is likely that the responses may have utility with more ethologically relevant sensory stimuli, but this possibility is not explored by the authors. For instance, the different behavioral options may be entirely driven by the weighting of the somatosensory maps that are driven by the sensory inputs which then drive the behaviors.

It is known that the different behaviors under investigation are more reliably triggered in response to localized mechanosensory stimuli. For instance, a rapid turn to the right is reliably elicited by a localized mechanosensory stimulus on the left side of the head. Crawling forward is reliably triggered by mechanosensory inputs to the posterior. Reversals are triggered by strong mechanosensory stimuli to the head while "hunching" may be triggered by lower sensory stimuli (these ideas are embodied in Maurice Kernan's touch scale (and the authors may want to cite Kernan)).

Given that the individual behaviors have utility in the context of localized mechanosensory stimuli, the uniform, body-wide, stimulus of the air puff and the probabilistic behavioral responses make more sense. The network is producing a random response to a weak mechanosensory stimulus that is likely to be received uniformly across the body. Alternatively, there may be stochastic variation in how the stimulus is received by an individual animal across trials and this could be reflected in the behavioral outcome.

Indeed, as the reviewer pointed out, decision-making is an ambiguous term that is used in different fields of biology to describe different processes that lead to making choices that do not necessarily involved neural network. From the neurotheological perspective decision-making has been investigated in species from simple invertebrate, including insects, worms etc. due to the tractability of the nervous system and its amenability to physiological recordings in most cases to vertebrates, fish, rodents, primates, including humans. The term decision-making has thus been used to describe many different types of decisions. It is true that most of these decisions involve processes like cost-benefits weighing. More recently the term

sensorimotor decision-making has been used to describe the processes of choosing a motor action based on a sensory stimulus) (Barker and Baier, 2015; Felsen and Mainen, 2012). However, the boundaries between these different categories of decisions or even definitions of decisions may often be blurry, as for example even sensorimotor decisions may involve evaluating a value of an option and therefore cost benefit weighing (Cisek, 2012). Given that it is unclear to which extent the findings from the studying the neural circuits of sensorimotor decisions will be relevant for other types of decision-making, we agree with the reviewer that the identification of candidate neurons in different regions of the nervous system for competitive interactions during sensorimotor decisions is not sufficient to conclude that decision-making in general is distributed and additional experimental evidence will be necessary to evaluate that claim. We therefore toned down or removed these statements throughout the text, especially in the results section (i.e. at the end of the section 2.5.) and discussion section where we now specifically claim that the findings of the candidate elements for competitive inetractions in the different areas of the nervous system are relevant to the response to the air-puff (l.780):

“Taken altogether, the GAL4 lines that we identified as hits drive in neurons that are located in the ventral nerve cord (both abdomen and thorax region), suboesophageal ganglion and brain (Fig. 8, S6 Fig., S7 Fig.). This suggests that the networks for competitive interactions between actions that occur in response to air-puff are distributed across the nervous system.”

We have clarified throughout the paper that all our findings, hypothesis and conclusions only are relevant for sensorimotor decisions, not decision-making in general.

To the point, that different individuals may receive stimuli somewhat differently although we can not exclude that some differences exist in how the stimulus is received, It is not very likely that these differences contribute significantly to the difference in type of responses selected as we have shown that the orientation of the larva with respect to the stimulus direction does not have an important effect on the type of action chosen (Fig S7, l.527-532).

We favor the idea that other factors like possible individual differences, internal state and the difference of different responses across trials may speak to a probabilistic exploratory element to find the most appropriate options. It will be indeed interesting to study how these decisions can be modulated by the external or internal context and whether some external or internal factor may bias the selection of the response.

We have cited Kernan’s work related to light touch sensing. We thank the reviewer for this remark and the reminder.

3.) In line 648 it is stated that the air puff was delivered continuously for 38 seconds and then there was a second delivery of 10 2-second-long air puffs. Then it is stated in line 652 that the analysis was performed on behavioral probabilities for “the first seconds after stimulus onset and the transition probabilities 3 seconds after stimulus onset. This sentence is unclear, as there are 11 different stimuli that were presented. Which stimulus was analyzed? The 38-second-long stimulus? All of the 2-second-

long stimuli?

In the air-puff assay used for screening the stimulus was applied for 38s continuously after 45s of monitoring the baseline larval behavior. Then after 22s after stimulus ended, ten 2s pulses of air-puff were delivered with an 8s interval between each pulse. We have designed such an assay to be also able to screen for neurons involved in habituation upon repeated presentation of the stimulus and response variability for example. However, for the purpose of this study we only quantified the actions during the initial response of larvae after the onset of the first 38s –long stimulus. We clarified that in the text of the material and methods. This paragraph on page 22 line 947-953 now is:

"The air-puff was delivered at the 45th second and applied for 38 seconds. After a period of recovery of 22 seconds when 10 air-puff pulses, 2 second each, were delivered (with a 8 second separation interval). We computed behavioral and transitional probabilities for different time-windows during stimulation. In this study, for the data from the behavioral screen, we show behavioral probabilities and transitions probabilities of the initial response of the larvae to the stimulus, immediately after the first stimulus onset. Specifically, we show the behavioral probabilities for the first second after the first stimulus onset and the transition probabilities for the first three seconds after stimulus onset. "

Minor Points:

Throughout the introduction, the authors refer to "the nervous system." It would be more accurate to write "this nervous system" or "nervous systems."

We have changed that when appropriate, for example in line 101, 106.

Figure 3 panel E NompC is misspelled as nomC. Both sides of scale bar in bottom left are labeled "decrease." It appears as if the scale to the right side should be labelled increase.

Thank you for noticing this. We have corrected this in the Figure 3

Figure 5A again has both sides of the color scale labeled as "decrease."

Thank you for noticing this. We have corrected this in the Figure 5

On page 10 line 317 please clarify the term "mechanosensory neurons." There are many different classes of mechanosensory neurons in the larval PNS.

We have corrected this and the sentence now reads: Interestingly in these sparse lines some of the neurons are downstream of mechanosensory chordotonal neurons (Jupiter, Hook-1, Baton, A27a),

some on the motor side (A19c, A27l, A03a, A02d) consistent with the role of these neurons in sensorimotor behaviors.

On page 26 line 849 please clarify the following sentence: "Implicitly, different individuals have implicit amplitude based limits to behavior." (JB)

We have clarified this sentence that was not clear and it now reads:

For example, different researchers have implicit "amplitude based" limits of behavior, *i.e.* two experts may diverge on a behavior being performed based on assumptions regarding amplitudes.

What is depicted in each of the panels in Figure S3? This cannot be understood from the legend or from the text.

We have clarified this on the legend and it now reads:

Inter-trial and inter-individual variability of mechanosensory responses
A-F. Ethogram of the behaviors of the attP2>TNT control before, and upon repeated presentation of 2 s air-puff stimulation. Each line is a larva. Different colors represent different actions: Blue-Hunch, Red-Bend, Cyan-Back-up, Black-Crawl and in Green- Stop **A-E.** Different examples of inter-trial variability (within each ethogram) and inter-individual variability (between different ethograms). **A**. Example of larvae that hunched on the first stimulus, bended on the second stimulus and performed any action on the third stimulus. **B**. Example of larvae that hunched on the first stimulus, performed a small action on the second stimulus and performed any action on a third stimulus. **C**. Example of larvae that bended on the first stimulus, hunched on the second stimulus and performed any action on the third stimulus **D**. Example of larvae that backed-up on the first stimulus, and performed any action on the second and third stimulus **E**. Example of larvae that performed a small action on the first stimulus, hunched on the second stimulus and performed any action on a third stimulus **F.** Example of larvae that only hunched immediately upon repeated presentation of an air-puff stimulus

Reviewer #3: This manuscript uses Drosophila larval behavior to address issues concerning behavioral choice, decision-making, and selection of different types of actions, such as sequential or mutually exclusive. They perform behavioral assays on how the animals move upon being faced with a puff of air, grouping them into specific actions such as hunch, bend, back-up and stop. This is done using a collection of gal-4 lines and TNT to inhibit synaptic transmission. The neurons are then matched with EM volume of the larval CNS.
My views are somewhat mixed on this paper, which may very well reflect my lack of expertise in the area. On one hand, I greatly appreciate the type of work the authors are attempting, and the work indeed will provide a wonderful foundation for future analysis. However, many of the points were either unclear or diffuse or too broad, such that it was difficult to get what the actual take home lessons from some of these experiments were. Thus, I will outline my views to what I found not so clear in the order the paper is preseted.

1. The beginning of the results was somewhat confusing, since they say they describe wildtype and those with inactivated neurons in terms of behavioral response, and refers to Figure 1A-B. But all of Figure 1 is on wild type. So a rewording would help. It would also help Figure 1 if the colors are explained in the figure itself and not just in the legends.

In the first section of the results (section 2.1), we describe only the behavioral responses of the intact larvae where no neurons have been inactivated. In order to make this clear, we have added a sentence at the beginning of the second paragraph: "We first characterized the response of intact larvae to a mechanical stimulus: air-puff. "

in Figure 1C we have changed the font color for each action so that it corresponds to the colors shown in the plots for Figure 1.

2. If I understand the assay properly, a group of larvae on a surface is faced with a puff of air. What is the distribution of the types of response (hunch, bend, etc.), based on the position of an animal within the surface relative to the source of stimulus? For example, from the Figure 1A, the air stream hits different animals at different positions, so do these have different outputs?

We thank the reviewer for this observation. In a previous study, to exclude the possibility that differences in the mechanical stimulus were at the origin of the different types of responses: Hunching or Bending, we drove Chrimson in the chordotonal sensory neurons which allows to uniformly activate all the chordotonals sensory neurons using optogenetics. Repeated optogenetic activation of chordotonals in the same animal resulted in both Hunching and Bending (Jovanic et al. 2016 (Figure S1).

In response to reviewer's comments and in order to show that the orientation of the animals with respect to the stimulus didn't have a significant effect on the type of response selected across the different five actions, we have plotted the probability distribution of the different action with respect to the angle representing the orientation of the larvae with respect to the direction of the airflow and found no significant differences as can be seen in Figure S8. This is described in more details in the material and method section:

"In order to question whether the air puff angle did play a role in behavior selection, we computed the cumulative distribution function (CDF) of the larva angles (for the control: w;;attP2-TNT larvae) when compared to the wind for all behavior when the stimulus started. Results are shown in figure S8. CDF curves did not exhibit specific patterns (safe from stopping action) suggesting preferred angle for decision making when related to the wind direction. We perform KS test to compare the main actions considered in this paper with: p=0.2 for Hunch-bend, p=0.18 for bend-back and p=0.04 for hunch-back."

3. In 2.2, there are three paragraphs (starting with line 171, 188 and 198) where they explain the

exceptions. It may help to clump all these at the end (or at once), since this breaks up the main positive message.

We have made this change and the paragraph that started at line 171 is now the last paragraph in the section 2.2.

4. In 2.4, there is the correlation between EM and light identified neurons, where an entire paragraph describes what has been done before. A similar thing occurs in 2.5, starting with line 347. It may help to have a table in the main figure which describes what has been done before and what is new in the current manuscript. For the latter, how indeed were these done, i.e. correlate the EM neurons with their flp-out and/or sparse lines? In a similar vein, what could explain the statement (line 373) that some could not be found in the EM column?

The information on anatomical annotations as well as the references for the EM reconstructions that show which neurons were reconstructed and identified before and which one in this paper can be found in Tables S8 and S9. However, the identification of these neurons in the driver lines from the screen and the description of the expression patterns of these driver lines to our knowledge have not been published previously (Figure 4, Figure 6, Table S8).

The correlation between EM and light microscopy images was done by matching the morphology (i.e. axon bouton, dendrite, and cell body positions) of the reconstructed neurons in Electron microscopy using the CATMAID software (Saalfeld et al., 2009; Schneider-Mizell et al., 2016) to the flp-out images of the GAL4 line that enables the visualize single neuron morphology. This is briefly described in the method section and has been use by us and others in previous studies (Jovanic et al., 2016; 2019).

In addition to the connectivity for the newly reconstructed neurons all the connectivity between the previously reconstructed neurons was not known because other studies focused on different circuits. For example, a neuron may have been reconstructed because it was a downstream partner of a neuron of interest in a study. But all its other presynaptic or postsynaptic partners were not described. In this study we systematically investigated all the synaptic connections between the neurons identified in the driver lines and some of the key neurons from our previous work involved in air-puff related behaviors (Jovanic et al., 2016; 2019). The synthesis of the connectivity in this network in Figure 8 and S6 was not known before.

To the reviewer's question why some of the neurons could not be found in the EM:

We have explored in the EM volume of the CNS the neurons that were reconstructed in the anatomical locations of the neurons identified in Light Microscopy and found neurons that were previously reconstruction or partially reconstructed (hook-1). In some cases, no neurons were reconstructed enough to unambiguously recognize and match them to the light microscopy images and time consuming exploratory and systematic reconstruction in order to find them in the EM volume were

beyond the scope of this study as it is the part of following up on the candidate neurons of interest found in the screen.

5. The reasoning for their last part is a bit unclear. Line 416 "...but only later. We therefore...". Since this is the final part, it may help to remind the people again.

We have clarified this section and it now reads this:

Sequence transition could also be affected in lines that were not among the best 25 hit lines for competitive interactions between Hunch/Bend/Back-up at the start of the sequence (just upon stimulus onset) but later during the response. Such lines would not come up in our competitive interaction hit lines that were chosen based on their phenotype in the first second upon stimulus onset.

6. In Discussion, page 15, I was a bit confused as what their main argument is, simply because it seems like every possibility is being discussed. For example, they use the terms like perceptual decision, early sensory processing, competitive interactions between different sensorimotor pathways. One can say yes, it could be involved in any one of these steps, but this is somewhat unsatisfying, because this goes almost into asking what process being observed, indeed, is NOT considered decision-making?

The listed hypothesis in a paragraph the reviewer pointed represents three different possibilities of the neural architecture underlying choice of a response to a mechanical stimulus. In the first one, it suggested that the circuit that we have previously identified that implements the choice between Hunch and Bend (Jovanic et al., 2016), could categorize the stimulus depending on some the stimulus properties or animal's state. In the second, it is the top down pathways who are the main contributors to the bias towards hunching or bending but modulating the activity in the circuit and finally in the last one there will be multiple sites for competitive interactions in the nervous system that would also contribute to the decision-making process. We favor the last one based on the fact that we have identified multiple candidates whose phenotypes are consistent with a role in competitive interactions.

Competitive interactions may underlie different types of processes: decision-making in terms of which behavior to perform or stimulus categorization. While the regions where these processes may occur can be different and may rely on different factors, the circuit mechanisms underlying competitive interactions may be shared. This was discussed in a recent review (Koyama and Pujala, 2018). So, competitive interactions that the candidate neurons seem to be involved may underlie different processes along the sensorimotor pathway including decision-making at different stages of processing. In addition, some of these processes may be intertwined.

We have simplified and clarified the idea of the distributed neural substrates for competitive interactions and the implication of that finding. We have removed the paragraph that was confusing in the discussion section. In addition, throughout the text we have made a number of changes to make the point clearer that the neuronal candidates in this paper are involved in competitive interactions during sensorimotor decisions and that the idea of a distributed mechanisms of making a choice may explain sensorimotor decisions specifically (not necessarily all types of decisions).

7. I personally found the mere description of the neurons (Figure 4 H-J) and Figure 6, although necessary, somewhat unhelpful. Perhaps an attempt could be made to make this part scientifically more relevant, e.g., presenting these in some sort of a circuit? Similarly, Figure 8 was extremely unhelpful. Yes, it describes the data, but perhaps a better subdivisions into conceptual units or pathways might help. I don't have any concrete suggestions, unfortunately, but a bit more effort here would be great for the general audience, I think.

We have made a simplified schematic of the connectivity within the mechanosensory network comprising the neurons shown in Figures 4 and 6. This representation shows the main connections and reveals the different parallel ascending pathways to higher regions of the nervous system downstream of both chordotonal and mdIII related interneuron. It also shows the pathways toward the premotor domain as well as the different feedback pathways within the network. This schematic is now Figure 8 and we have put the old Figure 8 in supplementary for more details representation of all the connections within the network.

In addition, we have simplified, clarified and shortened the descriptions of connectivity in sections 2.4. and 2.5 as well as provided more context for these descriptions.

References

Barker, A.J., and Baier, H. (2015). Sensorimotor decision making in the zebrafish tectum. Curr. Biol. *25*, 2804–2814.

Burgos, A., Honjo, K., Ohyama, T., Qian, C.S., Shin, G.J.-E., Gohl, D.M., Silies, M., Tracey, W.D., Zlatic, M., Cardona, A., et al. (2018). Nociceptive interneurons control modular motor pathways to promote escape behavior in Drosophila. eLife *7*, 1557.

Cisek, P. (2012). Making decisions through a distributed consensus. Current Opinion in Neurobiology *22*, 927–936.

Felsen, G., and Mainen, Z.F. (2012). Midbrain contributions to sensorimotor decision making. Journal of Neurophysiology *108*, 135–147.

Gerhard, S., Andrade, I., Fetter, R.D., Cardona, A., and Schneider-Mizell, C.M. (2017). Conserved neural circuit structure across Drosophila larval development revealed by comparative connectomics. eLife *6*, 2452.

Jovanic, T., Schneider-Mizell, C.M., Shao, M., Masson, J.-B., Denisov, G., Fetter, R.D., Mensh, B.D., Truman, J.W., Cardona, A., and Zlatic, M. (2016). Competitive Disinhibition Mediates Behavioral Choice and Sequences in Drosophila. Cell *167*, 858–870.e19.

Jovanic, T., Winding, M., Cardona, A., Truman, J.W., Gershow, M., and Zlatic, M. (2019). Neural

Substrates of Drosophila Larval Anemotaxis. Curr. Biol. *29*, 554–566.e554.

Ohyama, T., Schneider-Mizell, C.M., Fetter, R.D., Aleman, J.V., Franconville, R., Rivera-Alba, M., Mensh, B.D., Branson, K.M., Simpson, J.H., Truman, J.W., et al. (2015). A multilevel multimodal circuit enhances action selection in Drosophila. Nature *520*, 633–639.

Saalfeld, S., Cardona, A., Hartenstein, V., and Tomancak, P. (2009). CATMAID: collaborative annotation toolkit for massive amounts of image data. Bioinformatics *25*, 1984–1986.

Schneider-Mizell, C.M., Gerhard, S., Longair, M., Kazimiers, T., Li, F., Zwart, M.F., Champion, A., Midgley, F.M., Fetter, R.D., Saalfeld, S., et al. (2016). Quantitative neuroanatomy for connectomics in Drosophila. eLife *5*, 1133.

Takagi, S., Cocanougher, B.T., Niki, S., Miyamoto, D., Kohsaka, H., Kazama, H., Fetter, R.D., Truman, J.W., Zlatic, M., Cardona, A., et al. (2017). Divergent Connectivity of Homologous Command-like Neurons Mediates Segment-Specific Touch Responses in Drosophila. Neuron *96*, 1373–1387.e1376.

-We notice that you used “data not shown” and "unpublished data" in the manuscript ("not shown" in Fig 8 legend and "unpublished data" on pages 31 and mentioned in the Acknowledgments). PLOS Genetics recently updated its data access policy and now requires that all data be either published with the manuscript or made available in a publically accessible database. We ask that you either amend the supplementary material to include the referenced data or remove as many of the references as possible. Please include an explanation of how you have met this request (i.e. whether you have simply removed the instances because they are not integral to the manuscript, or whether you have provided further supporting information files) at the end of your response to reviewers. Please note that we will not be able to pass your manuscript through our technical checks unless this is accompanied by this explanation.

The weak connections not shown for clarity in Fig 8. This is commonly done in representations of network connectivity for visual clarity. All the connections are shown in the Supplementary connectivity matrix. We have clarified that in the legend of Fig 8.

We have removed the references to the unpublished data on page 31 as they are not necessary for this manuscript. I.e. data from activation screen for used to enrich the training for the roll behavior that was not addressed in this study.

In the acknowledgments section, we have removed the expression unpublished data as the said data are published in this study (EM reconstruction of hook-1)
